# Interval-Valued Random Matrices

**DOI:** 10.3390/e26110899

**Published:** 2024-10-23

**Authors:** Abdolnasser Sadeghkhani, Ali Sadeghkhani

**Affiliations:** 1 Department of Mathematics and Statistics, North Carolina Agricultural and Technical State University, Greensboro, NC 27411, USA; 2 Department of Mathematics and Statistics, University of Windsor, Windsor, ON N9B 3P4, Canada; sadeghk@uwindsor.ca

**Keywords:** Bayesian estimators, dominance, Frobenius norm loss function, interval-valued matrix, maximum likelihood estimator, AMS (2000) subject classification: Primary: 62C10, Secondary: 62F15, 62E15

## Abstract

This paper introduces a novel approach that combines symbolic data analysis with matrix theory through the concept of interval-valued random matrices. This framework is designed to address the complexities of real-world data, offering enhanced statistical modeling techniques particularly suited for large and complex datasets where traditional methods may be inadequate. We develop both frequentist and Bayesian methods for the statistical inference of interval-valued random matrices, providing a comprehensive analytical framework. We conduct extensive simulations to compare the performance of these methods, demonstrating that Bayesian estimators outperform maximum likelihood estimators under the Frobenius norm loss function. The practical utility of our approach is further illustrated through an application to climatology and temperature data, highlighting the advantages of interval-valued random matrices in real-world scenarios.

## 1. Introduction

Symbolic Data Analysis (SDA), first introduced by [1], focuses on analyzing complex data types that can encompass multiple values, distributions, or more generalized forms rather than traditional single-valued types. Unlike classical random variables, which take on a single, specific value, symbolic random variables can represent a range of values, leading to an internal distribution that captures uncertainty or variability within the data. The foundation of SDA relies on the concept that exploratory analyses and statistical inferences are often more meaningful at the group level rather than examining individual values in isolation [2].

SDA emphasizes the importance of group-level summaries—known as symbols—as the primary units of statistical analysis. These symbols, often represented as intervals, histograms, or other distributional forms that occupy a hypercube in $R^{p}$ (as opposed to considering values within $R^{p}$ single-valued data), serve as the foundation for statistical symbolic data [3,4].

Interval-valued data, a specific type of symbolic data, provide a structured way to represent information that naturally falls within ranges rather than precise point values. This approach has gained significant attention recently due to its ability to manage uncertainty and variability more effectively than traditional point-based methods. As discussed by [2,3,5], interval-valued data offer a versatile framework accommodating both univariate and multivariate forms, making them highly suitable for a wide range of real-world applications in various applied sciences.

The attractiveness of interval-valued data lies in their simplicity, flexibility, and broad applicability, driving extensive research on their statistical analysis. Recent studies have investigated both frequentist and Bayesian methods for constructing estimators tailored to interval-valued data (e.g., [5,6]. Moreover, innovative statistical techniques have been developed to explore relationships within interval-valued datasets. For example, ref. [7] introduced a Principal Component Analysis (PCA) method designed to represent intervals, while other methods have refined regression techniques to directly handle intervals. Some researchers have focused on extracting numerical characteristics from intervals, such as the midpoint [8], the midpoint and range [9], or the lower and upper limits [10], then applying these attributes to standard regression models. Alternatively, other researchers have chosen to work with intervals directly, treating them as the core unit of analysis without converting them into numerical summaries (e.g., [11,12]).

Complex data can also be studied in the context of random matrices, which extend the principles of univariate and multivariate analysis to matrix-based data structures. Unlike conventional multivariate methods that treat data as vectors, matrix-variate approaches are particularly well-suited for the analysis of data that naturally take the form of matrices, which are common in multidimensional settings. These methods are effective for the modeling of complex dependencies where multiple variables are inter-related and organized into rows and columns, capturing two-dimensional structures. Applications of matrix-variate random variables are found in various fields, such as econometrics, multivariate analysis, and machine learning, where they offer sophisticated approaches for the handling of relational data (see, e.g., [13], and references therein).

Matrix-variate methodologies offer significant advantages for the analysis of complex data, as they effectively capture correlations both within rows and across columns of a matrix. This ability allows for the simultaneous analysis of multiple variables while accounting for their interdependencies. Methods such as the use of matrix-variate normal distributions and matrix regression models provide robust frameworks for investigating relationships at both the row and column levels. This dual-level analysis is especially valuable in practical applications involving relational data, such as the joint analysis of multiple financial indicators in econometrics or understanding covariance structures among feature matrices in machine learning. For more information, see [14].

With the increasing complexity and dimensionality of modern datasets, both matrix-variate random variables and interval-valued data provide flexible frameworks to tackle these challenges. Interval-valued data handle uncertainty and variability by representing values as intervals rather than fixed points, effectively accommodating the imprecision common in real-world data. In contrast, matrix-variate methods are adept at modeling complex dependencies within matrix-structured data. Combining these two approaches—matrix-variate methods and interval-valued data—offers a powerful strategy for the analysis of large, complex datasets, allowing for a more comprehensive modeling of uncertainties and dependencies. This integration enhances the flexibility and robustness of statistical models, expanding their applicability to more intricate, high-dimensional scenarios.

This paper aims to explore the integration of matrix-variate and interval-valued data approaches through both frequentist and Bayesian methods. By examining how these methodologies can be combined, it seeks to develop a comprehensive strategy that enhances the analysis of complex data structures, accommodating both uncertainty and multidimensional dependencies. The focus is on leveraging the strengths of both methods—frequentist methods for their precision and objectivity and Bayesian methods for their flexibility and incorporation of prior knowledge—to create robust inferential frameworks. This dual approach is designed to be highly applicable across a range of scientific and practical fields, such as econometrics, machine learning, and bioinformatics, where complex and high-dimensional data are commonly encountered. Ultimately, this study aims to provide new insights and tools for researchers and practitioners to better understand, model, and interpret intricate datasets.

The rest of this paper is organized as follows. We begin with some preliminaries and definitions regarding matrix-variate and tensor distributions in Section 2. We continue by defining likelihood functions and Maximum Likelihood (ML) estimators, along with their asymptotic distributions. Section 3 is dedicated to defining priors, posteriors, and Bayes estimators under the Frobenius norm loss function, as well as the corresponding dominance results. In Section 4, we present simulations to demonstrate the superiority of the proposed estimators and to validate the theoretical results. Section 5 involves an analysis of temperature variations across different seasons using a dataset comprising interval-valued temperature matrices. Finally, concluding remarks are provided in Section 6.

## 2. Likelihood Function of Interval-Valued Matrices and ML Estimators

Before defining the likelihood function for interval-valued random variables, we introduce the tensor Wishart and inverse tensor Wishart distributions, which generalize the classical Wishart distribution. For a more detailed discussion, see [13,15].

**Definition** **1** (Tensor Wishart Distribution)**.** *Let* A *be a* p×p×q *positive definite tensor, where for each fixed* i∈{1,…,q}*, matrix slice* $A^{\left(i\right)}$ *is positive definite. We say that* A *follows a tensor Wishart distribution, as denoted by* ${\mathrm{TW}}_{p,q}\left(m,V\right)$*, where m represents the degrees of freedom and* V *is the scale tensor. The probability density function (PDF) is given by*

(1) $${\mathrm{TW}}_{p,q}\left(A|m,V\right)=\frac{\prod_{i=1}^{q}{\left|A^{\left(i\right)}\right|}^{\frac{m-p-1}{2}}}{2^{\frac{mpq}{2}}\prod_{i=1}^{q}{\left|V^{\left(i\right)}\right|}^{\frac{m}{2}}\Gamma_{p,q}\left(\frac{m}{2}\right)}\exp\left(-\frac{1}{2}\sum_{i=1}^{q}tr\left({\left(V^{\left(i\right)}\right)}^{-1}A^{\left(i\right)}\right)\right),$$

*where* $|A^{\left(i\right)}|$ *and* tr(·) *refer to the determinant and trace of matrix* $A^{\left(i\right)}$*, respectively. The generalized multivariate gamma function for tensors is denoted by* $\Gamma_{p,q}\left(\cdot \right)$ *and is expressed as*

$$\Gamma_{p,q}\left(\frac{m}{2}\right)=\pi^{\frac{pq(p-1)}{4}}\prod_{i=1}^{p}\prod_{j=1}^{q}\Gamma \left(\frac{m+1-i-j}{2}\right)$$

*The degrees of freedom must satisfy* m>p−1 *to ensure that each matrix slice (*$A^{\left(i\right)}$*) is invertible. The expectation of* A *is* E[A]=mV.

**Definition** **2** (Inverse Tensor Wishart Distribution)**.** *Let* $B=A^{-1}$*, where the inverse is defined slice-wise, i.e., for each i,* $B^{\left(i\right)}={\left(A^{\left(i\right)}\right)}^{-1}$*. Then,* B *follows an inverse tensor Wishart distribution, as denoted* ${\mathrm{ITW}}_{p,q}\left(m,U\right)$*, where* $U=V^{-1}$ *is the scale tensor. Its PDF is given by*

$${\mathrm{ITW}}_{p,q}\left(B|m,U\right)=\frac{\prod_{i=1}^{q}|U^{\left(i\right)}{|}^{\frac{m}{2}}\prod_{i=1}^{q}{\left|B^{\left(i\right)}\right|}^{-\frac{m+p+1}{2}}}{2^{\frac{mpq}{2}}\Gamma_{p,q}\left(\frac{m}{2}\right)}\exp\left(-\frac{1}{2}\sum_{i=1}^{q}\mathrm{tr}\left(U^{\left(i\right)}{\left(B^{\left(i\right)}\right)}^{-1}\right)\right),$$

*where* $|B^{\left(i\right)}|$ *and* tr(·) *refer to the determinant and trace of matrix* $B^{\left(i\right)}$*, respectively. The expectation of* B *is* $E\left[B\right]=\frac{U}{m-p-1}$*, which holds for* m>p+1*.*

In Definition 2, if U is a vector, then it corresponds to ${\mathrm{IW}}_{p}\left(m,U\right)$, and its PDF is given as

(2) $${\mathrm{IW}}_{p}\left(B|m,U\right)=\frac{{\left|U\right|}^{\frac{m}{2}}\;{\left|B\right|}^{-\frac{m+p+1}{2}}}{2^{\frac{mp}{2}}\Gamma_{p}\left(\frac{m}{2}\right)}\exp\left(-\frac{1}{2}\mathrm{tr}\left(UB^{-1}\right)\right)\;,$$

with E[B]=U/(m−p−1) for m>p+1.

To establish the structure of our model, we now consider the following interval-valued random matrix:

(3) $$\begin{array}{c} X=\left(\begin{array}{cccc} \left(a_{11},b_{11}\right) & \left(a_{12},b_{12}\right) & \ldots & \left(a_{1q},b_{1q}\right)\\ \left(a_{21},b_{21}\right) & \left(a_{22},b_{22}\right) & \ldots & \left(a_{2q},b_{2q}\right)\\ \vdots & \vdots & \ddots & \vdots \\ \left(a_{p1},b_{p1}\right) & \left(a_{p2},b_{p2}\right) & \ldots & \left(a_{pq},b_{pq}\right) \end{array}\right)\;, \end{array}$$

where each element forms an interval $\left(a_{jk},b_{jk}\right)$ with $a_{jk}<b_{jk}$ for j=1,…,p and k=1,…,q. This setup generalizes the concept of an interval-valued random vector of size p×1, which represents a hyper-rectangle (or box) in $R^{p}$. When X is a matrix of size p×q, where each element is an interval, the geometric interpretation becomes more complex. In this case, each row of the matrix can be viewed as a 1×q vector, with each element representing an interval in $R^{q}$. Consequently, each row represents a hyper-rectangle in $R^{q}$, and the entire matrix (X) can be interpreted as a collection or product of *p* such hyper-rectangles.

Overall, matrix X represents a generalized hyper-rectangle (or hyper-parallelepiped) in $R^{pq}$, where each interval contributes to the overall dimensionality of the object. This matrix structure introduces additional complexity by capturing variability across both rows and columns, resulting in a richer and more intricate geometric representation compared to the vector case.

Given that matrix X in (3) contains aggregated observed values over uniformly distributed intervals $\left(a_{jk},b_{jk}\right)$, with $a_{jk}<b_{jk}$ for j=1,…,p and k=1,…,q, we extend the results reported by [5,16,17] regarding the existence of a one-to-one correspondence between X and $\Theta ={\left(\Theta_{1},\Theta_{2}\right)}^{⊤}$, where $\Theta_{1}$ and $\Theta_{2}$ represent the mean matrix and the variance–covariance tensor of the internal distribution, respectively, and are expressed as

(4) $$\begin{array}{cc} \Theta_{1} & =\left(\begin{array}{cccc} \frac{a_{11}+b_{11}}{2} & \frac{a_{12}+b_{12}}{2} & \ldots & \frac{a_{1q}+b_{1q}}{2}\\ \frac{a_{21}+b_{21}}{2} & \frac{a_{22}+b_{22}}{2} & \ldots & \frac{a_{2q}+b_{2q}}{2}\\ \vdots & \vdots & \ddots & \vdots \\ \frac{a_{p1}+b_{p1}}{2} & \frac{a_{p2}+b_{p2}}{2} & \ldots & \frac{a_{pq}+b_{pq}}{2} \end{array}\right)\;, \end{array}$$

(5) $$\begin{array}{cc} \Theta_{2}=T_{ijkl} & =\left\{\begin{array}{cc} \frac{1}{12}{\left(b_{ij}-a_{ij}\right)}^{2}, & \mathrm{if}\;i=k\;\mathrm{and}\;j=l,\\ \frac{1}{12}\left(b_{ij}-a_{ij}\right)\left(b_{kl}-a_{kl}\right), & \mathrm{if}\;i\neq k\;\mathrm{or}\;j\neq l, \end{array}\right. \end{array}$$

where $a_{ij}<b_{ij}$ and $a_{kl}<b_{kl}$ for all i,k=1,…,p and j,l=1,…,q.

Let $X_{i}$ for i=1,…,n be a random matrix with the PDF denoted by $f_{i}^{X_{i}}\left(x_{i};\Theta \right)$. The associated parameters, $\Theta_{i1}$ and $\Theta_{i2}$, as defined in Equations (4) and (5), vary and take different values. The PDFs of these parameters are given by

(6) $$\begin{array}{cc} \Theta_{i1}\; & ∼{\mathrm{MN}}_{p\times q}\left(\mu ,\Sigma_{R},\Sigma_{C}\right)\;, \end{array}$$

(7) $$\begin{array}{cc} \Theta_{i2}\; & ∼{\mathrm{TW}}_{p,q}\left(m,V\right)\;, \end{array}$$

where ${\mathrm{MN}}_{p\times q}$ represents the matrix normal distribution with a mean matrix (μ) of size p×q, a row covariance matrix ($\Sigma_{R}$) of size p×p, and a column covariance matrix ($\Sigma_{C}$) of size q×q with the following PDF:

(8) $${\mathrm{MN}}_{p\times q}\left(\Theta_{1}|\mu ,\Sigma_{R},\Sigma_{C}\right)\;=\;\frac{1}{{\left(2\pi \right)}^{\frac{pq}{2}}\det{\left(\Sigma_{R}\right)}^{\frac{p}{2}}\det{\left(\Sigma_{C}\right)}^{\frac{q}{2}}}\mathrm{etr}\left\{-\frac{1}{2}\Psi^{-1}{\left(\Theta_{1}-\mu \right)}^{⊤}\Sigma_{R}^{-1}\left(\Theta_{1}-\mu \right)\right\}\;,$$

and ${\mathrm{TW}}_{p,q}$ represents the tensor Wishart distribution with degrees of freedom *m* and scale tensor V as in (1).

### 2.1. ML Estimators and Related Properties

For a given tensor (V), which can be decomposed into a product of matrices (${\left\{V^{\left(k\right)}\right\}}_{k=1}^{q}$), we introduce the *determinantal product*, which is defined as the product of the determinants of these matrices as follows:

(9) $$D\left(V\right)=\prod_{k=1}^{q}\left|V^{\left(k\right)}\right|,$$

where $|V^{\left(k\right)}|$ denotes the determinant of matrix $V^{\left(i\right)}$. Assuming that ${\left\{\Theta_{i1}\right\}}_{i=1}^{n}$ and ${\left\{\Theta_{i2}\right\}}_{i=1}^{n}$ are independent, the likelihood is given by $L=L_{1}\times L_{2}$, where

(10) $$\begin{array}{cc} L_{1}\;= & \;L_{1}\left(\mu ,\Sigma_{R},\Sigma_{C}|\Theta_{11},\ldots ,\Theta_{n1}\right)\;=\;|\Sigma_{R}{|}^{-\frac{nq}{2}}{\left|\Sigma_{C}\right|}^{-\frac{np}{2}}\\ \; & \times \exp\left(-\frac{1}{2}\sum_{i=1}^{n}\mathrm{tr}\left(\Sigma_{R}^{-1}\left(\Theta_{i1}-\mu \right)\Sigma_{C}^{-1}{\left(\Theta_{i1}-\mu \right)}^{⊤}\right)\right)\\ = & \;|\Sigma_{R}{|}^{-\frac{nq}{2}}{\left|\Sigma_{C}\right|}^{-\frac{np}{2}}\exp\left(-\frac{1}{2}\mathrm{tr}\left(S_{R}\Sigma_{R}^{-1}\right)\mathrm{tr}\left(S_{C}\Sigma_{C}^{-1}\right)\right)\;, \end{array}$$

(11) $$\begin{array}{cc} L_{2}\;= & \;L_{2}\left(V|\Theta_{12},\ldots ,\Theta_{n2}\right)=D{\left(V\right)}^{-\frac{nm}{2}}\exp\left(-\frac{1}{2}\sum_{i=1}^{n}\sum_{k=1}^{q}\mathrm{tr}\left(\Theta_{i2}^{\left(k\right)}{\left(V^{\left(k\right)}\right)}^{-1}\right)\right)\;\;, \end{array}$$

where

$$\begin{array}{cc} S_{R} & =\sum_{i=1}^{n}\left(\Theta_{i1}-{\bar{\Theta }}_{1}\right){\left(\Theta_{i1}-{\bar{\Theta }}_{1}\right)}^{⊤}\;,\\ S_{C} & =\sum_{i=1}^{n}{\left(\Theta_{i1}-{\bar{\Theta }}_{1}\right)}^{⊤}(\Theta_{i1}-{\bar{\Theta }}_{1}),\\ {\bar{\Theta }}_{1} & =\frac{1}{n}\sum_{i=1}^{n}\Theta_{i1}\;. \end{array}$$

The ML estimators of the unknown mean matrix (μ), the covariance matrices ($\Sigma_{R}$ and $\Sigma_{C}$), and the tensor (V) are presented in the following theorem.

**Theorem** **1.** *The maximum likelihood (ML) estimators for the matrix parameters (*μ*,* $\Sigma_{R}$*, and* $\Sigma_{C}$*) and the tensor parameter (*V*) are given by*

(12) $$\begin{array}{cc} {\hat{\mu }}^{ML} & ={\bar{\Theta }}_{1}\;, \end{array}$$

(13) $$\begin{array}{cc} {\hat{\Sigma }}_{R}^{ML} & =\frac{S_{R}}{nq}\;, \end{array}$$

(14) $$\begin{array}{cc} {\hat{\Sigma }}_{C}^{ML} & =\frac{S_{C}}{np}\;, \end{array}$$

(15) $$\begin{array}{cc} {\hat{V}}^{ML} & =\left(\frac{\sum_{i=1}^{n}\Theta_{i2}^{\left(1\right)}}{nm},\frac{\sum_{i=1}^{n}\Theta_{i2}^{\left(2\right)}}{nm},\ldots ,\frac{\sum_{i=1}^{n}\Theta_{i2}^{\left(q\right)}}{nm}\right). \end{array}$$

**Proof.**  To find the ML estimator for μ, we differentiate the log-likelihood function ($\log L_{1}$) with respect to μ and set it to zero as follows:

$$\begin{array}{cc} \frac{\partial \log L_{1}}{\partial \mu } & =\sum_{i=1}^{n}\Sigma_{R}^{-1}\left(\Theta_{i1}-\mu \right)\Sigma_{C}^{-1}=0\;. \end{array}$$

Solving for μ, we obtain (12). For $\Sigma_{R}$ and $\Sigma_{C}$, we differentiate $\log L_{1}$ with respect to $\Sigma_{R}$ and $\Sigma_{C}$ and solve the resulting equations, yielding (13) and (14), respectively.

For the tensor parameter (V), we differentiate the log-likelihood function ($\log L_{2}$) with respect to each slice ($V^{\left(k\right)}$) of V, set it to zero, and solve

$$\frac{\partial \log L_{2}}{\partial V^{\left(k\right)}}=-\frac{nm}{2}{\left(V^{\left(k\right)}\right)}^{-1}+\frac{1}{2}{\left(V^{\left(k\right)}\right)}^{-1}\sum_{i=1}^{n}\Theta_{i2}^{\left(k\right)}{\left(V^{\left(k\right)}\right)}^{-1}=0\;,\;\mathrm{for}\;k=1,\ldots ,q.$$

Multiplying by $V^{\left(k\right)}$, rearranging terms, and solving for $V^{\left(k\right)}$, we obtain

$${\hat{V}}^{(k),ML}=\frac{\sum_{i=1}^{n}\Theta_{i2}^{\left(k\right)}}{nm}\;,\;\mathrm{for}\;k=1,\ldots ,q.$$

Thus, the MLE for the tensor parameter V is given by

$${\hat{V}}^{ML}=\left(\frac{\sum_{i=1}^{n}\Theta_{i2}^{\left(1\right)}}{nm},\frac{\sum_{i=1}^{n}\Theta_{i2}^{\left(2\right)}}{nm},\ldots ,\frac{\sum_{i=1}^{n}\Theta_{i2}^{\left(q\right)}}{nm}\right).$$

□

### 2.2. Asymptotic Properties of ML Estimators

**Theorem** **2.** *Consider* Sym(p,q)*, the set of* p×p *and* q×q *real symmetric matrices, and let* P(p,q)⊆Sym(p,q) *represent the subset consisting of symmetric positive definite matrices, forming convex regular cones. Setting* $\omega =\left(\mu ,\Sigma_{R},\Sigma_{C}\right)\in \Omega =R^{p\times q}\times P\left(p\right)\times P\left(q\right)$*, we have*

$$\begin{array}{c} \sqrt{n}\left(\hat{\omega }-\omega \right)\overset{d}{\to }{\mathrm{MN}}_{m}\left(0_{m},I^{-1}\left(\omega \right)\right)\;, \end{array}$$

*where* I(ω) *is the Fisher information matrix with elements given by*

$$\begin{array}{c} I_{ij}\left(\omega \right)\;=\;{\left[\frac{\partial \mu }{\partial \omega_{i}}\right]}^{⊤}\Sigma_{R}^{-1}\frac{\partial \mu }{\partial \omega_{j}}+\frac{1}{2}\mathrm{tr}\left(\Sigma_{R}^{-1}\frac{\partial \Sigma_{R}}{\partial \omega_{i}}\Sigma_{R}^{-1}\frac{\partial \Sigma_{R}}{\partial \omega_{j}}\right)+\frac{1}{2}\mathrm{tr}\left(\Sigma_{C}^{-1}\frac{\partial \Sigma_{C}}{\partial \omega_{i}}\Sigma_{C}^{-1}\frac{\partial \Sigma_{C}}{\partial \omega_{j}}\right)\;, \end{array}$$

*and* $m=\dim\left(\Omega \right)=pq+\frac{p(p+1)}{2}+\frac{q(q+1)}{2}$.

**Proof.**  The Fisher information matrix for matrix-variate distributions (see, e.g., [13]) is more precisely characterized by the covariance tensor of the score (gradient of the log-likelihood function). Specifically, if we let ${\mathrm{MN}}_{p,q}\left(\Theta_{1}|\mu ,\Sigma_{R},\Sigma_{C}\right)$ denote the matrix normal distribution as in (8), then the Fisher information matrix is given by

$$\begin{array}{cc} I(\omega ) & ={\mathrm{Cov}}_{\omega }\left[\nabla \log{\mathrm{MN}}_{p,q}\left(\Theta_{1}|\mu ,\Sigma_{R},\Sigma_{C}\right)\right]\\ \; & =E_{\omega }\left[\nabla \log{\mathrm{MN}}_{p,q}\left(\Theta_{1}|\mu ,\Sigma_{R},\Sigma_{C}\right)⊗\nabla \log{\mathrm{MN}}_{p,q}{\left(\Theta_{1}|\mu ,\Sigma_{R},\Sigma_{C}\right)}^{⊤}\right]\\ \; & =-E_{\omega }\left[\nabla^{2}\log{\mathrm{MN}}_{p,q}\left(\Theta_{1}|\mu ,\Sigma_{R},\Sigma_{C}\right)\right]. \end{array}$$

Here, the expectation is assumed with respect to the distribution parameterized by $\omega =(\mu ,\Sigma_{R},\Sigma_{C})$. Note that the covariance is represented in terms of a tensor product, emphasizing that the covariance structure involves interactions between the matrices.

Following the theory presented by [18,19], the Fisher information matrix for the matrix normal distribution can be broken down using the Kronecker product properties of the row and column covariances. For matrix-variate distributions parameterized by an *m*-dimensional vector ($\psi =\left(\psi_{1},\ldots ,\psi_{m}\right)\in R^{m}$, where $\mu =(\psi_{1},\ldots ,\psi_{pq})$ and $\Sigma_{R}\left(\psi \right)$ and $\Sigma_{C}\left(\psi \right)$ are the row and column covariance matrices corresponding to the remaining parameters), the Fisher information matrix components are given by

$$\begin{array}{c} I_{ij}\left(\omega \right)\;=\;{\left[\frac{\partial \mu }{\partial \omega_{i}}\right]}^{⊤}\Sigma_{R}^{-1}\frac{\partial \mu }{\partial \omega_{j}}+\frac{1}{2}\mathrm{tr}\left(\Sigma_{R}^{-1}\frac{\partial \Sigma_{R}}{\partial \omega_{i}}\Sigma_{R}^{-1}\frac{\partial \Sigma_{R}}{\partial \omega_{j}}\right)+\frac{1}{2}\mathrm{tr}\left(\Sigma_{C}^{-1}\frac{\partial \Sigma_{C}}{\partial \omega_{i}}\Sigma_{C}^{-1}\frac{\partial \Sigma_{C}}{\partial \omega_{j}}\right). \end{array}$$

These components follow from the matrix differentiation results that account for the second-order derivatives and trace operations needed to properly capture the covariance interactions.

To verify that the stationary point corresponds to a maximum of the log-likelihood function, we conduct a second derivative test by examining the Hessian matrix of the negative log-likelihood function at the stationary point. If this Hessian is negative definite, it confirms that the point is a maximum. Given that the Fisher information matrix (I(ω)) reflects the curvature of the log-likelihood function, its negative definiteness guarantees that our stationary point is, indeed, a local maximum.

Therefore, the Fisher information matrix for the matrix normal distribution respects the Kronecker product structure and tensor properties of the distribution, as detailed by [13,19]. This completes the proof. □

## 3. Bayesian Setup

In a Bayesian setup, we begin by defining independent prior distributions for the unknown matrix and tensor parameters in models (6) and (7). Using the determinantal product defined in (9), the prior distribution is given by

(16) $$\pi \left(\mu ,\Sigma_{R},\Sigma_{C},V\right)\propto |\Sigma_{R}{|}^{-\frac{p+2}{2}}{\left|\Sigma_{C}\right|}^{-\frac{q+2}{2}}D{\left(V\right)}^{-\frac{pq+1}{2}},$$

where μ is the mean matrix; $\Sigma_{R}$ and $\Sigma_{C}$ are the row and column covariance matrices, respectively; and V represents the tensor of interest.

Given the independent likelihood functions in (10) and (11), the joint posterior distribution of the matrices ($\mu ,\Sigma_{R},\Sigma_{C}$) and the tensor (V) can be written as

(17) $$\begin{array}{cc} \pi (\mu ,\Sigma_{R},\Sigma_{C},V|\Theta ) & \propto L_{1}\left(\mu ,\Sigma_{R},\Sigma_{C}|\Theta_{11},\ldots ,\Theta_{1n}\right)\;L_{2}\left(V|\Theta_{12},\ldots ,\Theta_{n2}\right)\;\pi \left(\mu ,\Sigma_{R},\Sigma_{C},V\right)\\ \; & \propto |\Sigma_{R}{|}^{-\frac{nq+p+2}{2}}{\left|\Sigma_{C}\right|}^{-\frac{np+q+2}{2}}\\ \; & \times \exp\left(-\frac{1}{2}\mathrm{tr}\left(S_{R}\Sigma_{R}^{-1}\right)-\frac{1}{2}\mathrm{tr}\left(S_{C}\Sigma_{C}^{-1}\right)\right)\\ \; & \times \exp\left(-\frac{n}{2}\sum_{i=1}^{n}\mathrm{tr}\left({\left(\Theta_{i1}-\mu \right)}^{⊤}\Sigma_{R}^{-1}\left(\Theta_{i1}-\mu \right)\Sigma_{C}^{-1}\right)\right)\\ \; & \times D{\left(V\right)}^{-\frac{pq+1+nmpq}{2}}\exp\left(-\frac{1}{2}\sum_{i=1}^{n}\sum_{k=1}^{q}\mathrm{tr}\left(\Theta_{i2}^{\left(k\right)}{\left(V^{\left(k\right)}\right)}^{-1}\right)\right). \end{array}$$

where $S_{R}$ and $S_{C}$ are the scatter matrices for row and column covariances, respectively.

The following lemma provides the full conditional posterior distributions associated with the posterior distribution in (17).

**Lemma** **1.** *The full conditional distributions associated with the posterior distribution in (17) are given by*

(18) $$\begin{array}{cc} \mu |\Sigma_{R},\Sigma_{C},\Theta_{1} & ∼{\mathrm{MN}}_{p,q}\left({\bar{\Theta }}_{1},\frac{\Sigma_{R}}{n},\frac{\Sigma_{C}}{n}\right), \end{array}$$

(19) $$\begin{array}{cc} \Sigma_{R}|\mu ,\Sigma_{C},\Theta_{1} & ∼{\mathrm{IW}}_{p}\left(nq+1,S_{R}+\sum_{i=1}^{n}\left(\Theta_{i1}-\mu \right)\Sigma_{C}^{-1}{\left(\Theta_{i1}-\mu \right)}^{⊤}\right), \end{array}$$

(20) $$\begin{array}{cc} \Sigma_{C}|\mu ,\Sigma_{R},\Theta_{1} & ∼{\mathrm{IW}}_{q}\left(np+1,S_{C}+\sum_{i=1}^{n}\left(\Theta_{i1}^{⊤}-\mu^{⊤}\right)\Sigma_{R}^{-1}\left(\Theta_{i1}^{⊤}-\mu^{⊤}\right)\right), \end{array}$$

(21) $$\begin{array}{cc} V|\Theta_{2} & ∼{\mathrm{ITW}}_{p,q}\left(nm,\sum_{i=1}^{n}\Theta_{i2}\right), \end{array}$$

*where* ${\mathrm{MN}}_{p,q}$ *is the matrix normal distribution,* ${\mathrm{IW}}_{p}$ *and* ${\mathrm{IW}}_{q}$ *are inverse Wishart distributions, and* ${\mathrm{ITW}}_{pq}$ *is the inverse tensor Wishart distribution.*

**Proof.**  From the joint posterior distribution in (17), it can easily be seen that

$$L\left(\mu |\Sigma_{R},\Sigma_{C},\Theta_{1}\right)\propto \exp\left(-\frac{n}{2}\sum_{i=1}^{n}{\left(\Theta_{i1}-\mu \right)}^{⊤}\Sigma_{R}^{-1}\left(\Theta_{i1}-\mu \right)\Sigma_{C}^{-1}\right)\;;$$

thus, the full conditional distribution for μ is

$$\mu |\Sigma_{R},\Sigma_{C},\Theta_{1}∼{\mathrm{MN}}_{p,q}\left({\bar{\Theta }}_{1},\Sigma_{R}/n,\Sigma_{C}/n\right),$$

where ${\bar{\Theta }}_{1}=\frac{1}{n}\sum_{i=1}^{n}\Theta_{i1}$ is the sample mean.

Next, the full conditional for $\Sigma_{R}$ comes from the part of the posterior involving $\Sigma_{R}$

$$L\left(\Sigma_{R}|\mu ,\Sigma_{C},\Theta_{1}\right)\propto {\left|\Sigma_{R}\right|}^{-\frac{nq+p+2}{2}}\exp\left(-\frac{1}{2}\mathrm{tr}\left(S_{R}\Sigma_{R}^{-1}\right)\right),$$

where $S_{R}=\sum_{i=1}^{n}\left(\Theta_{i1}-\mu \right)\Sigma_{C}^{-1}{\left(\Theta_{i1}-\mu \right)}^{⊤}$. This corresponds to an inverse Wishart distribution in (2), so we have

$$\Sigma_{R}|\mu ,\Sigma_{C},\Theta_{1}∼{\mathrm{IW}}_{p}\left(nq+1,S_{R}\right).$$

Similarly, the full conditional for $\Sigma_{C}$ is derived as

$$L\left(\Sigma_{C}|\mu ,\Sigma_{R},\Theta_{1}\right)\propto {\left|\Sigma_{C}\right|}^{-\frac{np+q+2}{2}}\exp\left(-\frac{1}{2}\mathrm{tr}\left(S_{C}\Sigma_{C}^{-1}\right)\right),$$

where $S_{C}=\sum_{i=1}^{n}\left(\Theta_{i1}^{⊤}-\mu^{⊤}\right)\Sigma_{R}^{-1}\left(\Theta_{i1}^{⊤}-\mu^{⊤}\right)$. Hence, the full conditional for $\Sigma_{C}$ is

$$\Sigma_{C}|\mu ,\Sigma_{R},\Theta_{1}∼{\mathrm{IW}}_{q}\left(np+1,S_{C}\right).$$

Finally, the full conditional for V is derived from the likelihood involving V in model (7). This likelihood is proportional to an inverse tensor Wishart distribution (Definition 2). Specifically,

$$L\left(V|\Theta_{2}\right)\propto {\left|V\right|}^{-\frac{nm+pq+1}{2}}\exp\left(-\frac{1}{2}\;\mathrm{tr}\left(\sum_{i=1}^{n}\Theta_{i2}V^{-1}\right)\right),$$

which leads to the full conditional for V as in (21). □

### 3.1. Loss Functions

One of the most common loss functions for estimating a matrix (μ) using $\hat{\mu }$ is the Frobenius norm, which is given by

(22) $$L_{2}=||\mu -\hat{\mu }{\left|\right|}_{F}^{2}\;,$$

where, for a given matrix ($A\in R^{p\times q}$), the Frobenius norm is defined as

$${\left||A|\right|}_{F}=\sqrt{\sum_{i=1}^{p}\sum_{j=1}^{q}{\left|a_{ij}\right|}^{2}},$$

with $a_{ij}$ representing the elements of matrix A. The Frobenius norm can be interpreted as the square root of the sum of the squares of all the entries in the matrix. It corresponds to the Euclidean norm when the matrix is flattened into a vector. For more information, see [20,21].

For the tensor-valued case, the tensor Frobenius norm generalizes the matrix Frobenius norm. If M and $\hat{M}$ are tensors of the same dimensions, the loss function is given by

(23) $$L_{2}\left(M,\hat{M}\right)=||M-\hat{M}{\left|\right|}_{F}^{2},$$

where the tensor Frobenius norm for a tensor ($(M\in R^{p\times q\times r}$) is defined as

$${\left||M|\right|}_{F}=\sqrt{\sum_{i=1}^{p}\sum_{j=1}^{q}\sum_{k=1}^{r}{\left|m_{ijk}\right|}^{2}}.$$

The interpretation is similar to that of the matrix case; it is the square root of the sum of the squares of all the tensor elements. This generalization is crucial for the extension of matrix-based methods to higher-order data structures such as tensors.

### 3.2. Bayes Estimators

**Theorem** **3.** *Consider models (6) and (7) and prior (16). The Bayes estimators of matrix parameters* μ*,* $\Sigma_{R}$*, and* $\Sigma_{C}$ *(with respect to the Frobenius norm loss in (22)) and tensor* V *(with respect to the entropy loss function in (23)) are given by*

(24) $$\begin{array}{cc} {\hat{\mu }}^{B} & ={\bar{\Theta }}_{1}\;, \end{array}$$

(25) $$\begin{array}{cc} {\hat{\Sigma }}_{R}^{B} & =\frac{S_{R}}{nq+p}\;, \end{array}$$

(26) $$\begin{array}{cc} {\hat{\Sigma }}_{C}^{B} & =\frac{S_{C}}{np+q}\;, \end{array}$$

(27) $$\begin{array}{cc} {\hat{V}}^{B} & =\left(\frac{\sum_{i=1}^{n}\Theta_{i2}^{\left(1\right)}}{nm-p-1},\frac{\sum_{i=1}^{n}\Theta_{i2}^{\left(2\right)}}{nm-p-1},\ldots ,\frac{\sum_{i=1}^{n}\Theta_{i2}^{\left(q\right)}}{nm-p-1}\right). \end{array}$$

**Proof.**  The Bayes estimators are derived from the expectations of the posterior marginal distributions of the parameters. Based on the posterior distribution (18), one can easily show that the posterior marginal distribution for μ is

(28) $$\begin{array}{cc} \mu |\Theta & ∼{\mathrm{MT}}_{p,q}\left({\bar{\Theta }}_{1},\frac{S_{R}}{n+q},\frac{S_{C}}{n+p},n+q-p\right), \end{array}$$

where ${\mathrm{MT}}_{p,q}\left(M,\Sigma_{R},\Sigma_{C},\nu \right)$ represents a matrix T distribution with a mean matrix (M), row covariance matrix ($\Sigma_{R}$), column covariance matrix ($\Sigma_{C}$), and ν degrees of freedom, with the PDF given by

(29) $$\begin{array}{cc} {\mathrm{MT}}_{p,q}\left(\mu |M,\Sigma_{R},\Sigma_{C},\nu \right)= & \frac{\Gamma_{p}\left(\frac{\nu +q}{2}\right)}{{\left(\pi \right)}^{pq/2}\Gamma_{p}\left(\frac{\nu }{2}\right)\det{\left(\Sigma_{R}\right)}^{q/2}\det{\left(\Sigma_{C}\right)}^{p/2}}\\ & \times {\left|I_{p}+\left(\mu -M\right)\Sigma_{C}^{-1}{\left(\mu -M\right)}^{⊤}\Sigma_{R}^{-1}\right|}^{-(\nu +q)/2}. \end{array}$$

The expectation of the matrix T distribution in (29) is $E\left[\mu \right]=M={\bar{\Theta }}_{1}$, yielding (24).

Next, the posterior marginal distribution for $\Sigma_{R}$ is

(30) $$\begin{array}{cc} \Sigma_{R}|\Theta & ∼{\mathrm{IW}}_{p}\left(nq+p,S_{R}\right), \end{array}$$

where ${\mathrm{IW}}_{p}$ is the inverse Wishart distribution. The expectation of the inverse Wishart distribution is $\frac{S_{R}}{nq+p}$, which proves (25).

Similarly, it can be shown that

(31) $$\begin{array}{cc} \Sigma_{C}|\Theta & ∼{\mathrm{IW}}_{q}\left(np+q,S_{C}\right), \end{array}$$

Hence, the proof of (26) is complete.

To derive the Bayes estimator for the tensor parameter (V), note that the posterior distribution for V given as $\Theta_{i2}$ follows an inverse tensor Wishart distribution as follows:

$$V|\Theta_{i2}∼{\mathrm{ITW}}_{p,q}\left(nm,\sum_{i=1}^{n}\Theta_{i2}\right)\;,$$

and the expectation of the inverse tensor Wishart distribution is

$$E\left[V|\Theta_{i2}\right]=\frac{\sum_{i=1}^{n}\Theta_{i2}}{nm-p-1}\;,$$

which completes the proof for (27). □

In statistical estimation, evaluating the efficacy of Bayes estimators compared to ML estimators is crucial, especially under non-informative priors. The next theorem investigates the performance of Bayes estimators under a proposed prior distribution for matrix and tensor parameters compared to ML estimators. Our goal is to demonstrate that, under the Frobenius norm loss function, Bayes estimators exhibit dominance over their ML counterparts.

**Theorem** **4.** *Under the assumptions of Theorem 3, the Bayes estimators of parameters* $\Sigma_{R}$*,* $\Sigma_{C}$*, and* V*given in Equations (25)–(27) dominate the ML estimators obtained by Theorem 1.*

**Proof.**  To prove that the Bayes estimators dominate the ML estimators under the Frobenius norm loss, we need to compute the differences in risk between the Bayes and ML estimators for the matrix parameters ($\Sigma_{R}$ and $\Sigma_{C}$) and the tensor parameter (V). These differences in risk are given by the following expected losses:

(32) $$\begin{array}{cc} \Delta_{\Sigma_{R}} & =E\left[L_{2}\left({\hat{\Sigma }}_{R}^{ML},\Sigma_{R}\right)-L_{2}\left({\hat{\Sigma }}_{R}^{B},\Sigma_{R}\right)\right], \end{array}$$

(33) $$\begin{array}{cc} \Delta_{\Sigma_{C}} & =E\left[L_{2}\left({\hat{\Sigma }}_{C}^{ML},\Sigma_{C}\right)-L_{2}\left({\hat{\Sigma }}_{C}^{B},\Sigma_{C}\right)\right], \end{array}$$

(34) $$\begin{array}{cc} \Delta_{V} & =E\left[L_{2}\left({\hat{V}}^{ML},V\right)-L_{2}\left({\hat{V}}^{B},V\right)\right]. \end{array}$$

For the matrix parameter ($\Sigma_{R}$), the ML and Bayes estimators are given by (13) and (25), respectively. The expected loss (under Frobenius norm squared) for the ML estimator is

$$E\left[L_{2}\left({\hat{\Sigma }}_{R}^{ML},\Sigma_{R}\right)\right]=\frac{\mathrm{tr}(\Sigma_{R}^{2})}{nq},$$

and for the Bayes estimator, we have

$$E\left[L_{2}\left({\hat{\Sigma }}_{R}^{B},\Sigma_{R}\right)\right]=\frac{\mathrm{tr}(\Sigma_{R}^{2})}{nq+p}.$$

Therefore,

$$\Delta_{\Sigma_{R}}=\frac{\mathrm{tr}(\Sigma_{R}^{2})}{nq}-\frac{\mathrm{tr}(\Sigma_{R}^{2})}{nq+p}=\mathrm{tr}\left(\Sigma_{R}^{2}\right)\left(\frac{1}{nq}-\frac{1}{nq+p}\right).$$

Since $\frac{1}{nq}>\frac{1}{nq+p}$, it follows that $\Delta_{\Sigma_{R}}>0$.

Similarly, using the ML and Bayes estimators given by (14) and (26), respectively, we have

$$E\left[L_{2}\left({\hat{\Sigma }}_{C}^{ML},\Sigma_{C}\right)\right]=\frac{\mathrm{tr}(\Sigma_{C}^{2})}{np},$$

and

$$E\left[L_{2}\left({\hat{\Sigma }}_{C}^{B},\Sigma_{C}\right)\right]=\frac{\mathrm{tr}(\Sigma_{C}^{2})}{np+q}.$$

Thus, the difference in risk is

$$\Delta_{\Sigma_{C}}=\frac{\mathrm{tr}(\Sigma_{C}^{2})}{np}-\frac{\mathrm{tr}(\Sigma_{C}^{2})}{np+q}=\mathrm{tr}\left(\Sigma_{C}^{2}\right)\left(\frac{1}{np}-\frac{1}{np+q}\right)\;.$$

Since $\frac{1}{np}>\frac{1}{np+q}$, it follows that $\Delta_{\Sigma_{C}}>0$.

For the tensor parameter (V), the ML and Bayes estimators are given by (15) and (27), respectively. The expected loss for the ML estimator is

$$E\left[L_{2}\left({\hat{V}}^{ML},V\right)\right]=\frac{D(V)}{nm},$$

where $D\left(V\right)=\mathrm{tr}\left(V^{2}\right)=\sum_{i,j,k}V_{ijk}^{2}$. For the Bayes estimator, it is

$$E\left[L_{2}\left({\hat{V}}^{B},V\right)\right]=\frac{D(V)}{nm-pq-1}.$$

Thus, the difference in risk is

$$\Delta_{V}=\frac{D(V)}{nm}-\frac{D(V)}{nm-pq-1}=D\left(V\right)\left(\frac{1}{nm}-\frac{1}{nm-pq-1}\right)$$

Since $\frac{1}{nm}>\frac{1}{nm-pq-1}$, it follows that $\Delta_{V}>0$.

Therefore, the Bayes estimators for $\Sigma_{R}$, $\Sigma_{C}$, and V dominate the ML estimators under the Frobenius norm loss. □

## 4. Simulation Results

In this section, we present simulation results to evaluate the performance of the ML estimators (Equations (12)–(15)) and Bayesian estimators (Equations (24)–(27)) for interval-valued matrix data under models (6) and (7). The parameters of interest include the row covariance matrix ($\Sigma_{R}$), the column covariance matrix ($\Sigma_{C}$), and the variance–covariance tensor (V). To assess the performance of these estimators, we compute the expected loss (also known as risk) under the Frobenius norm loss functions given by (22) and (23) for matrix and tensor estimators, respectively.

The simulation setup involves generating N=1000 iterations with sample sizes of n=25,100, and 250 of interval-valued matrices with known matrix and tensor parameters, as shown below.

$$\begin{array}{c} \mu =\left(\begin{array}{ccc} 1 & 0 & 0\\ 0 & 1 & 0\\ 0 & 0 & 1 \end{array}\right),\;\Sigma_{R}=\left(\begin{array}{ccc} 1.5 & 0 & 0\\ 0 & 1.5 & 0\\ 0 & 0 & 1.5 \end{array}\right),\;\Sigma_{C}=\left(\begin{array}{ccc} 2 & 0 & 0\\ 0 & 2 & 0\\ 0 & 0 & 2 \end{array}\right), \end{array}$$

$$V\left(:,:,1\right)=\left(\begin{array}{ccc} 1.5 & 0 & 0\\ 0 & 1.5 & 0\\ 0 & 0 & 1.5 \end{array}\right),\;V\left(:,:,2\right)=\left(\begin{array}{ccc} 2 & 0 & 0\\ 0 & 2 & 0\\ 0 & 0 & 2 \end{array}\right),\;V\left(:,:,3\right)=\left(\begin{array}{ccc} 2.5 & 0 & 0\\ 0 & 2.5 & 0\\ 0 & 0 & 2.5 \end{array}\right).$$

It can be observed that p=q=3. The ML and Bayesian estimations of the parameters, with *m* selected as n−pq−1, for each sample size are provided in Table 1, Table 2 and Table 3.

Table 4 illustrates the risk functions of the ML and Bayes estimators under the Frobenius norm loss for different sample sizes (n=25,100,250). In Table 4, we observe that for small sample sizes (n=25), the Bayesian estimator generally outperforms the maximum likelihood (ML) estimator, particularly for the row and column covariance matrices ($\Sigma_{R}$ and $\Sigma_{C}$). As the sample size increases to n=100 and beyond, the risk values for both ML and Bayes estimators decrease, indicating improved performance. Additionally, the difference between the risks of the two estimators narrows, showing that their performances become more similar. This convergence is due to the consistency property of ML estimators, which ensures asymptotically good performance. As more data become available, the influence of prior information in the Bayesian approach diminishes, leading both estimators to yield similar results.

## 5. Real Application Example

The dataset consists of interval-valued (minimum and maximum) temperature matrices representing temperature variations across different seasons—winter, spring, summer, and fall—i.e., p=4, over a 20-year period spanning four time intervals, namely 1950–1969, 1970–1989, 1990–2009, and 2010–2023, (q=4), in n=39 Asian countries.

In other words, for each country, the data are arranged in a matrix format where the rows correspond to seasons and the columns correspond to different time periods spanning multiple years. Each matrix element is an interval representing the minimum and maximum temperatures recorded during the corresponding period. The data are sourced from the Berkeley Earth Surface Temperature Study and can be accessed at Kaggle. Table 5 illustrates the dataset’s structure.

Table 6 presents the ML and Bayesian estimations for matrix parameters μ, $\Sigma_{R}$, and $\Sigma_{C}$. Table 7, Table 8, Table 9 and Table 10 provide estimations for the tensor parameter (V), with each table showing one season’s component of the tensor. Note that *m* is set to n−pq−1.

While we observed the superiority of Bayes estimators over ML estimators for the matrix parameters (μ and $\Sigma_{R}$) and the tensor parameter (V), we further assessed the difference in the average length of the corresponding 95% intervals for each element of the parameters. Specifically, we calculated the average length of the 95% confidence intervals obtained from the ML estimation as $l^{ML}=\frac{1}{16}\sum_{i=1}^{4}\sum_{j=1}^{4}\left(U^{ML}\left(C_{ij}\right)-L^{ML}\left(C_{ij}\right)\right)$ and, similarly, the average length of the 95% credible intervals from the Bayesian estimation as $l^{B}=\frac{1}{16}\sum_{i=1}^{4}\sum_{j=1}^{4}\left(U^{B}\left(C_{ij}\right)-L^{B}\left(C_{ij}\right)\right)$. Here, $U^{ML}$ and $U^{B}$ represent the upper limits of the corresponding intervals for element $C_{ij}$, while $L^{ML}$ and $L^{B}$ represent the lower limits. Each parameter matrix can be denoted generically as $C=(C_{ij})$, for i,j=1,…,4. The results indicate that the average length of the credible intervals from the Bayesian estimation is consistently shorter than that of the ML confidence intervals, confirming that Bayesian estimators provide narrower intervals. This finding is consistent with our previous simulation results, which demonstrated the superior performance of Bayesian estimation.

## 6. Concluding Remarks

In this study, we introduced the concept of interval-valued random matrices, representing a significant advancement at the intersection of symbolic data analysis and matrix theory. This novel approach offers a robust framework for statistical modeling, particularly in contexts characterized by complex and large datasets where traditional methods may fall short.

One of the strengths of our model is its ability to leverage both frequentist and Bayesian approaches for statistical inference. We have demonstrated that, especially when the sample size is small, Bayesian estimators dominate maximum likelihood estimators under the Frobenius norm loss function. Moreover, the asymptotic distribution of the ML estimators was established. This work paves the way for further research and application across various fields, encouraging the exploration of similar frameworks to handle symbolic data. The use of non-informative priors enhances decision making in complex analytical scenarios, ultimately improving the quality of statistical modeling in diverse disciplines.

A critical application explored in this paper involves analyzing temperature variations across different seasons using a dataset of interval-valued temperature matrices. Specifically, we examined seasonal temperature ranges (winter, spring, summer, and fall) across four distinct 20-year periods from 1950 to 2023. This practical example underscores the versatility of interval-valued random matrices, demonstrating their capacity to capture and analyze variability in temperature data.

## Figures and Tables

**Table 1:** ML and Bayes estimations of the mean matrix (μ), row covariance matrix ($\Sigma_{R}$), column covariance matrix ($\Sigma_{C}$), and tensor (V) for n=25.

Parameter	ML Estimator	Bayes Estimator
μ	$\left(\begin{array}{ccc} 0.9933 & 0.0105 & -0.0122\\ -0.0067 & 0.9869 & 0.0091\\ 0.0024 & -0.0034 & 0.9886 \end{array}\right)$	$\left(\begin{array}{ccc} 0.9933 & 0.0105 & -0.0122\\ -0.0067 & 0.9869 & 0.0091\\ 0.0024 & -0.0034 & 0.9886 \end{array}\right)$
$\Sigma_{R}$	$\left(\begin{array}{ccc} 2.9068 & 0.0003 & -0.0140\\ 0.0003 & 2.8717 & -0.0097\\ -0.0140 & -0.0097 & 2.9047 \end{array}\right)$	$\left(\begin{array}{ccc} 2.7950 & 0.0003 & -0.0135\\ 0.0003 & 2.76133 & -0.0093\\ -0.0135 & -0.0093 & 2.7930 \end{array}\right)$
$\Sigma_{C}$	$\left(\begin{array}{ccc} 2.8833 & -0.0065 & 0.0118\\ -0.0065 & 2.8976 & 0.0136\\ 0.0118 & 0.0136 & 2.902 \end{array}\right)$	$\left(\begin{array}{ccc} 2.7724 & -0.0063 & 0.0113\\ -0.0063 & 2.7861 & 0.0131\\ 0.0113 & 0.0131 & 2.790 \end{array}\right)$
V (ML Estimator)	$\left(\begin{array}{ccc} 1.4954 & -0.0020 & -0.0018\\ -0.0020 & 1.4996 & 0.0057\\ -0.0018 & 0.0057 & 1.5041 \end{array}\right)$	$\left(\begin{array}{ccc} 1.57413 & -0.0021 & -0.0019\\ -0.0021 & 1.5786 & 0.0060\\ -0.0019 & 0.0060 & 1.58336 \end{array}\right)$
	$\left(\begin{array}{ccc} 2.00304 & -0.0049 & -0.00096\\ -0.00490 & 1.9991 & -0.0076\\ -0.00096 & -0.0076 & 1.99592 \end{array}\right)$	$\left(\begin{array}{ccc} 2.1084 & -0.0051 & -0.0010\\ -0.0051 & 2.1043 & -0.0080\\ -0.0010 & -0.0080 & 2.1009 \end{array}\right)$
	$\left(\begin{array}{ccc} 2.49098 & -0.0092 & -0.0063\\ -0.0092 & 2.50823 & 0.0065\\ -0.0063 & 0.0065 & 2.4846 \end{array}\right)$	$\left(\begin{array}{ccc} 2.6220 & -0.0097 & -0.0067\\ -0.0097 & 2.6402 & 0.0068\\ -0.0067 & 0.0068 & 2.61544 \end{array}\right)$

**Table 2:** Estimated mean, row covariance, and column covariance matrices and tensor (V) for ML and Bayes estimators with n=100.

Parameter	ML Estimator	Bayes Estimator
μ	$\left(\begin{array}{ccc} 0.9938 & -0.0016 & -0.0034\\ -0.0055 & 1.0046 & -0.0010\\ -0.0086 & 0.0103 & 0.9996 \end{array}\right)$	$\left(\begin{array}{ccc} 0.9938 & -0.0016 & -0.0034\\ -0.0055 & 1.0046 & -0.0010\\ -0.0086 & 0.0103 & 0.9996 \end{array}\right)$
$\Sigma_{R}$	$\left(\begin{array}{ccc} 2.9673 & 0.0022 & 0.0046\\ 0.0022 & 2.9692 & 0.0014\\ 0.0046 & 0.0014 & 2.9567 \end{array}\right)$	$\left(\begin{array}{ccc} 2.9379 & 0.0022 & 0.0046\\ 0.0022 & 2.9398 & 0.0014\\ 0.0046 & 0.0014 & 2.9275 \end{array}\right)$
$\Sigma_{C}$	$\left(\begin{array}{ccc} 2.9806 & -0.0031 & 0.0107\\ -0.0031 & 2.9527 & 0.0051\\ 0.0107 & 0.0051 & 2.9600 \end{array}\right)$	$\left(\begin{array}{ccc} 2.9511 & -0.0030 & 0.0106\\ -0.0030 & 2.9234 & 0.0051\\ 0.0106 & 0.0051 & 2.9307 \end{array}\right)$
V (ML Estimator)	$\left(\begin{array}{ccc} 1.5019 & -0.0022 & -0.0033\\ -0.0022 & 1.4982 & -0.0000\\ -0.0033 & -0.0000 & 1.4985 \end{array}\right)$	$\left(\begin{array}{ccc} 1.5209 & -0.0022 & -0.0033\\ -0.0022 & 1.5172 & -0.0000\\ -0.0033 & -0.0000 & 1.5175 \end{array}\right)$
	$\left(\begin{array}{ccc} 2.0025 & 0.0038 & 0.0006\\ 0.0038 & 2.0016 & -0.0044\\ 0.0006 & -0.0044 & 1.9977 \end{array}\right)$	$\left(\begin{array}{ccc} 2.0278 & 0.0038 & 0.0007\\ 0.0038 & 2.0270 & -0.0045\\ 0.0007 & -0.0045 & 2.0230 \end{array}\right)$
	$\left(\begin{array}{ccc} 2.5010 & -0.0020 & -0.0034\\ -0.0020 & 2.4972 & 0.0028\\ -0.0034 & 0.0028 & 2.4986 \end{array}\right)$	$\left(\begin{array}{ccc} 2.5327 & -0.0020 & -0.0034\\ -0.0020 & 2.5288 & 0.0028\\ -0.0034 & 0.0028 & 2.5303 \end{array}\right)$

**Table 3:** Estimated mean, row covariance, and column covariance matrices and tensor (V) for ML and Bayes estimators with n=250.

Parameter	ML Estimator	Bayes Estimator
μ	$\left(\begin{array}{ccc} 1.0016 & -0.0013 & -0.0031\\ -0.0020 & 0.9988 & -0.0031\\ 0.0017 & 0.0003 & 1.0044 \end{array}\right)$	$\left(\begin{array}{ccc} 1.0016 & -0.0013 & -0.0031\\ -0.0020 & 0.9988 & -0.0031\\ 0.0017 & 0.0003 & 1.0044 \end{array}\right)$
$\Sigma_{R}$	$\left(\begin{array}{ccc} 2.9975 & 0.0046 & -0.0008\\ 0.0046 & 2.9846 & 0.0019\\ -0.0008 & 0.0019 & 2.9909 \end{array}\right)$	$\left(\begin{array}{ccc} 2.9856 & 0.0046 & -0.0008\\ 0.0046 & 2.9727 & 0.0019\\ -0.0008 & 0.0019 & 2.9790 \end{array}\right)$
$\Sigma_{C}$	$\left(\begin{array}{ccc} 2.9942 & -0.0006 & 0.0025\\ -0.0006 & 2.9927 & -0.0064\\ 0.0025 & -0.0064 & 2.9862 \end{array}\right)$	$\left(\begin{array}{ccc} 2.9823 & -0.0006 & 0.0025\\ -0.0006 & 2.9808 & -0.0064\\ 0.0025 & -0.0064 & 2.9743 \end{array}\right)$
V (ML Estimator)	$\left(\begin{array}{ccc} 1.4999 & 0.0011 & -0.0013\\ 0.0011 & 1.4994 & -0.0008\\ -0.0013 & -0.0008 & 1.4998 \end{array}\right)$	$\left(\begin{array}{ccc} 1.5075 & 0.0011 & -0.0013\\ 0.0011 & 1.5069 & -0.0008\\ -0.0013 & -0.0008 & 1.5073 \end{array}\right)$
	$\left(\begin{array}{ccc} 2.0010 & -0.0019 & -0.0004\\ -0.0019 & 2.0005 & -0.0022\\ -0.0004 & -0.0022 & 1.9979 \end{array}\right)$	$\left(\begin{array}{ccc} 2.0110 & -0.0019 & -0.0004\\ -0.0019 & 2.0106 & -0.0022\\ -0.0004 & -0.0022 & 2.0079 \end{array}\right)$
	$\left(\begin{array}{ccc} 2.4966 & 0.0002 & 0.0004\\ 0.0002 & 2.4988 & -0.0007\\ 0.0004 & -0.0007 & 2.5007 \end{array}\right)$	$\left(\begin{array}{ccc} 2.5092 & 0.0002 & 0.0004\\ 0.0002 & 2.5113 & -0.0007\\ 0.0004 & -0.0007 & 2.5133 \end{array}\right)$

**Table 4:** Comparison of risks for ML and Bayes estimators under Frobenius norm loss for different sample sizes (n=25,100,250).

Sample Size (n)	Parameter	ML Risk	Bayes Risk
25	Mean Matrix, μ	0.1180	0.1180
	Row Covariance, $\Sigma_{R}$	0.8050	0.6938
	Column Covariance, $\Sigma_{C}$	0.4211	0.3472
	Tensor, V	0.0379	0.0346
100	Mean Matrix, μ	0.0301	0.0301
	Row Covariance, $\Sigma_{R}$	0.7544	0.7253
	Column Covariance, $\Sigma_{C}$	0.3489	0.3295
	Tensor, V	0.0072	0.0069
250	Mean Matrix, μ	0.0122	0.0122
	Row Covariance, $\Sigma_{R}$	0.7569	0.7450
	Column Covariance, $\Sigma_{C}$	0.3437	0.3358
	Tensor, V	0.0028	0.0028

**Table 5:** Interval-valued matrices for selected Asian countries.

Country	Interval-Valued Matrix
United Arab Emirates	$\left(\begin{array}{cccc} \left(15.75,21.5\right) & \left(16.08,21.97\right) & \left(17.1,23.56\right) & \left(19.24,22.1\right)\\ \left(21.43,30.25\right) & \left(19.79,31.43\right) & \left(19.8,32.34\right) & \left(22.49,32.73\right)\\ \left(31.06,34.42\right) & \left(30.53,34.92\right) & \left(31.36,36.1\right) & \left(33.34,36.37\right)\\ \left(23.09,32.53\right) & \left(22.7,32.44\right) & \left(23.7,33.34\right) & \left(24.95,34.47\right) \end{array}\right)$
Iraq	$\left(\begin{array}{cccc} \left(4.72,14.98\right) & \left(6.42,15.09\right) & \left(6.64,15.55\right) & \left(9.66,14.89\right)\\ \left(12.98,30.34\right) & \left(14.14,30.29\right) & \left(13.06,31.3\right) & \left(15.38,30.88\right)\\ \left(30.89,36.43\right) & \left(31.46,36.58\right) & \left(31.91,38.28\right) & \left(33.8,37.9\right)\\ \left(15.36,32.06\right) & \left(13.36,33.37\right) & \left(15.46,32.97\right) & \left(14.24,33.64\right) \end{array}\right)$
Azerbaijan	$\left(\begin{array}{cccc} \left(-5.15,5.53\right) & \left(-5.91,5.94\right) & \left(-4.11,5.74\right) & \left(-2.42,7.26\right)\\ \left(0.72,18.24\right) & \left(0.59,17.58\right) & \left(2.38,17.55\right) & \left(3.08,19.09\right)\\ \left(18.58,25.34\right) & \left(18.01,25.89\right) & \left(18.91,26.51\right) & \left(22.01,27\right)\\ \left(2.44,21.27\right) & \left(4.93,20.78\right) & \left(1.7,21.25\right) & \left(2.83,20.68\right) \end{array}\right)$
⋮	⋮
Turkey	$\left(\begin{array}{cccc} \left(-6.2,5.74\right) & \left(-5.99,5.57\right) & \left(-5.37,4.98\right) & \left(-3.27,5.25\right)\\ \left(-0.44,16.88\right) & \left(-0.39,16.45\right) & \left(2.09,18.3\right) & \left(2.54,17.46\right)\\ \left(16.86,23.66\right) & \left(17.29,24.22\right) & \left(18.02,25.75\right) & \left(18.3,26.04\right)\\ \left(2.76,20.23\right) & \left(2.64,19.35\right) & \left(2.79,21.36\right) & \left(1.54,20.92\right) \end{array}\right)$

**Table 6:** ML and Bayes estimators of parameters.

Estimator	Value
${\hat{\mu }}^{ML}$	$\left(\begin{array}{cccc} 13.52397 & 13.88910 & 14.34654 & 14.70949\\ 21.54192 & 21.82295 & 22.27026 & 22.78782\\ 27.19256 & 27.46731 & 27.90397 & 28.41500\\ 22.57231 & 22.44821 & 22.52013 & 22.62744 \end{array}\right)$
${\hat{\Sigma }}_{R}^{ML}$	$\left(\begin{array}{cccc} 0.109389249 & 0.072021087 & 0.075759802 & -0.007602738\\ 0.072021087 & 0.103560670 & 0.081688315 & 0.007713221\\ 0.075759802 & 0.081688315 & 0.087399746 & 0.004339049\\ -0.007602738 & 0.007713221 & 0.004339049 & 0.064772075 \end{array}\right)$
${\hat{\Sigma }}_{C}^{ML}$	$\left(\begin{array}{cccc} 11.69090 & 11.52679 & 11.48719 & 11.59250\\ 11.52679 & 11.40047 & 11.37008 & 11.47697\\ 11.48719 & 11.37008 & 11.38523 & 11.48793\\ 11.59250 & 11.47697 & 11.48793 & 11.69355 \end{array}\right)$
${\hat{\mu }}^{B}$	$\left(\begin{array}{cccc} 13.52397 & 13.88910 & 14.34654 & 14.70949\\ 21.54192 & 21.82295 & 22.27026 & 22.78782\\ 27.19256 & 27.46731 & 27.90397 & 28.41500\\ 22.57231 & 22.44821 & 22.52013 & 22.6274 \end{array}\right)$
${\hat{\Sigma }}_{R}^{B}$	$\left(\begin{array}{cccc} 0.106654518 & 0.070220560 & 0.073865807 & -0.007412669\\ 0.070220560 & 0.100971654 & 0.079646107 & 0.007520391\\ 0.073865807 & 0.079646107 & 0.085214753 & 0.004230573\\ -0.007412669 & 0.007520391 & 0.004230573 & 0.06315277 \end{array}\right)$
${\hat{\Sigma }}_{C}^{B}$	$\left(\begin{array}{cccc} 11.39863 & 11.23862 & 11.20001 & 11.30269\\ 11.23862 & 11.11546 & 11.08583 & 11.19004\\ 11.20001 & 11.08583 & 11.10060 & 11.20073\\ 11.30269 & 11.19004 & 11.20073 & 11.40121 \end{array}\right)$

**Table 7:** Winter season components of tensor $\hat{V}$—ML and Bayes.

Component	ML	Bayes
1	$\left(\begin{array}{cccc} 5.8586 & 3.5697 & 1.4356 & 3.4064\\ 5.7615 & 3.5114 & 1.4250 & 3.3843\\ 5.7587 & 3.4333 & 1.4111 & 3.4909\\ 5.8290 & 3.3441 & 1.3603 & 3.4502 \end{array}\right)$	$\left(\begin{array}{cccc} 5.8586 & 3.5697 & 1.4357 & 3.4064\\ 5.7616 & 3.5115 & 1.4250 & 3.3843\\ 5.7588 & 3.4334 & 1.4112 & 3.4910\\ 5.8291 & 3.3441 & 1.3604 & 3.4502 \end{array}\right)$
2	$\left(\begin{array}{cccc} 5.7615 & 3.5134 & 1.4234 & 3.3467\\ 5.6749 & 3.4612 & 1.4126 & 3.3270\\ 5.6719 & 3.3797 & 1.3979 & 3.4283\\ 5.7308 & 3.2955 & 1.3468 & 3.3975 \end{array}\right)$	$\left(\begin{array}{cccc} 5.7616 & 3.5134 & 1.4234 & 3.3467\\ 5.6750 & 3.4612 & 1.4127 & 3.3271\\ 5.6720 & 3.3797 & 1.3980 & 3.4283\\ 5.7309 & 3.2955 & 1.3469 & 3.3975 \end{array}\right)$
3	$\left(\begin{array}{cccc} 5.6447 & 3.4498 & 1.3978 & 3.2844\\ 5.5596 & 3.3979 & 1.3865 & 3.2654\\ 5.5601 & 3.3169 & 1.3723 & 3.3646\\ 5.6169 & 3.2380 & 1.3233 & 3.3359 \end{array}\right)$	$\left(\begin{array}{cccc} 5.7588 & 3.5196 & 1.4261 & 3.3508\\ 5.6720 & 3.4666 & 1.4146 & 3.3314\\ 5.6725 & 3.3840 & 1.4000 & 3.4326\\ 5.7305 & 3.3034 & 1.3500 & 3.4034 \end{array}\right)$
4	$\left(\begin{array}{cccc} 5.7136 & 3.4873 & 1.4195 & 3.3417\\ 5.6173 & 3.4270 & 1.4092 & 3.3178\\ 5.6169 & 3.3542 & 1.3983 & 3.4252\\ 5.6984 & 3.2683 & 1.3522 & 3.3810 \end{array}\right)$	$\left(\begin{array}{cccc} 5.8291 & 3.5578 & 1.4481 & 3.4093\\ 5.7309 & 3.4962 & 1.4377 & 3.3848\\ 5.7305 & 3.4220 & 1.4266 & 3.4944\\ 5.8136 & 3.3343 & 1.3795 & 3.4494 \end{array}\right)$

**Table 8:** Spring season components of tensor $\hat{V}$—ML and Bayes.

Component	ML	Bayes
1	$\left(\begin{array}{cccc} 3.4990 & 2.3897 & 1.0933 & 2.2433\\ 3.4438 & 2.3550 & 1.0885 & 2.2276\\ 3.4498 & 2.2949 & 1.0815 & 2.2852\\ 3.4873 & 2.2522 & 1.0387 & 2.2500 \end{array}\right)$	$\left(\begin{array}{cccc} 3.5697 & 2.4380 & 1.1154 & 2.2887\\ 3.5134 & 2.4026 & 1.1105 & 2.2726\\ 3.5196 & 2.3413 & 1.1033 & 2.3314\\ 3.5578 & 2.2977 & 1.0597 & 2.2955 \end{array}\right)$
2	$\left(\begin{array}{cccc} 3.4419 & 2.3550 & 1.0709 & 2.1986\\ 3.3926 & 2.3274 & 1.0677 & 2.1840\\ 3.3979 & 2.2642 & 1.0583 & 2.2380\\ 3.4270 & 2.2224 & 1.0143 & 2.2075 \end{array}\right)$	$\left(\begin{array}{cccc} 3.5115 & 2.4026 & 1.0926 & 2.2430\\ 3.4612 & 2.3744 & 1.0892 & 2.2282\\ 3.4666 & 2.3099 & 1.0797 & 2.2833\\ 3.4962 & 2.2674 & 1.0348 & 2.2521 \end{array}\right)$
3	$\left(\begin{array}{cccc} 3.3653 & 2.2949 & 1.0405 & 2.1529\\ 3.3128 & 2.2642 & 1.0380 & 2.1360\\ 3.3169 & 2.2090 & 1.0306 & 2.1924\\ 3.3542 & 2.1651 & 0.9880 & 2.1552 \end{array}\right)$	$\left(\begin{array}{cccc} 3.4334 & 2.3413 & 1.0616 & 2.1964\\ 3.3797 & 2.3099 & 1.0590 & 2.1792\\ 3.3840 & 2.2537 & 1.0515 & 2.2368\\ 3.4220 & 2.2089 & 1.0080 & 2.1987 \end{array}\right)$
4	$\left(\begin{array}{cccc} 3.2779 & 2.2522 & 1.0390 & 2.1153\\ 3.2303 & 2.2224 & 1.0354 & 2.1011\\ 3.2380 & 2.1651 & 1.0292 & 2.1531\\ 3.2683 & 2.1311 & 0.9891 & 2.1221 \end{array}\right)$	$\left(\begin{array}{cccc} 3.3441 & 2.2977 & 1.0600 & 2.1580\\ 3.2955 & 2.2674 & 1.0564 & 2.1436\\ 3.3034 & 2.2089 & 1.0500 & 2.1966\\ 3.3343 & 2.1742 & 1.0091 & 2.1650 \end{array}\right)$

**Table 9:** Summer Season Components of Tensor $\hat{V}$—ML and Bayes.

Component	ML	Bayes
1	$\left(\begin{array}{cccc} 1.4072 & 1.0933 & 0.7229 & 1.0710\\ 1.3952 & 1.0709 & 0.7211 & 1.0684\\ 1.3978 & 1.0405 & 0.7313 & 1.0896\\ 1.4195 & 1.0390 & 0.7112 & 1.0777 \end{array}\right)$	$\left(\begin{array}{cccc} 1.4357 & 1.1154 & 0.7375 & 1.0927\\ 1.4234 & 1.0926 & 0.7357 & 1.0900\\ 1.4261 & 1.0616 & 0.7461 & 1.1116\\ 1.4481 & 1.0600 & 0.7256 & 1.0995 \end{array}\right)$
2	$\left(\begin{array}{cccc} 1.3968 & 1.0885 & 0.7211 & 1.0657\\ 1.3847 & 1.0677 & 0.7212 & 1.0624\\ 1.3865 & 1.0380 & 0.7313 & 1.0835\\ 1.4092 & 1.0354 & 0.7106 & 1.0695 \end{array}\right)$	$\left(\begin{array}{cccc} 1.4250 & 1.1105 & 0.7357 & 1.0872\\ 1.4127 & 1.0892 & 0.7358 & 1.0839\\ 1.4146 & 1.0590 & 0.7461 & 1.1054\\ 1.4377 & 1.0564 & 0.7249 & 1.0911 \end{array}\right)$
3	$\left(\begin{array}{cccc} 1.3832 & 1.0815 & 0.7313 & 1.0669\\ 1.3703 & 1.0583 & 0.7313 & 1.0631\\ 1.3723 & 1.0306 & 0.7442 & 1.0856\\ 1.3983 & 1.0292 & 0.7248 & 1.0696 \end{array}\right)$	$\left(\begin{array}{cccc} 1.4112 & 1.1033 & 0.7461 & 1.0884\\ 1.3980 & 1.0797 & 0.7461 & 1.0846\\ 1.4000 & 1.0515 & 0.7592 & 1.1076\\ 1.4266 & 1.0500 & 0.7394 & 1.0912 \end{array}\right)$
4	$\left(\begin{array}{cccc} 1.3334 & 1.0387 & 0.7112 & 1.0322\\ 1.3202 & 1.0143 & 0.7106 & 1.0296\\ 1.3233 & 0.9880 & 0.7248 & 1.0508\\ 1.3522 & 0.9891 & 0.7102 & 1.0359 \end{array}\right)$	$\left(\begin{array}{cccc} 1.3604 & 1.0597 & 0.7256 & 1.0531\\ 1.3469 & 1.0348 & 0.7249 & 1.0504\\ 1.3500 & 1.0080 & 0.7394 & 1.0720\\ 1.3795 & 1.0091 & 0.7246 & 1.0568 \end{array}\right)$

**Table 10:** Fall season components of tensor $\hat{V}$—ML and Bayes.

Component	ML	Bayes
1	$\left(\begin{array}{cccc} 3.3389 & 2.2433 & 1.0710 & 2.2138\\ 3.2804 & 2.1986 & 1.0657 & 2.1976\\ 3.2844 & 2.1529 & 1.0669 & 2.2691\\ 3.3417 & 2.1153 & 1.0322 & 2.2290 \end{array}\right)$	$\left(\begin{array}{cccc} 3.4064 & 2.2887 & 1.0927 & 2.2586\\ 3.3467 & 2.2430 & 1.0872 & 2.2420\\ 3.3508 & 2.1964 & 1.0884 & 2.3149\\ 3.4093 & 2.1580 & 1.0531 & 2.2741 \end{array}\right)$
2	$\left(\begin{array}{cccc} 3.3173 & 2.2276 & 1.0684 & 2.1976\\ 3.2611 & 2.1840 & 1.0624 & 2.1877\\ 3.2654 & 2.1360 & 1.0631 & 2.2556\\ 3.3178 & 2.1011 & 1.0296 & 2.2232 \end{array}\right)$	$\left(\begin{array}{cccc} 3.3843 & 2.2726 & 1.0900 & 2.2420\\ 3.3271 & 2.2282 & 1.0839 & 2.2319\\ 3.3314 & 2.1792 & 1.0846 & 2.3012\\ 3.3848 & 2.1436 & 1.0504 & 2.2681 \end{array}\right)$
3	$\left(\begin{array}{cccc} 3.4218 & 2.2852 & 1.0896 & 2.2691\\ 3.3604 & 2.2380 & 1.0835 & 2.2556\\ 3.3646 & 2.1924 & 1.0856 & 2.3390\\ 3.4252 & 2.1531 & 1.0508 & 2.3041 \end{array}\right)$	$\left(\begin{array}{cccc} 3.4910 & 2.3314 & 1.1116 & 2.3149\\ 3.4283 & 2.2833 & 1.1054 & 2.3012\\ 3.4326 & 2.2368 & 1.1076 & 2.3863\\ 3.4944 & 2.1966 & 1.0720 & 2.3507 \end{array}\right)$
4	$\left(\begin{array}{cccc} 3.3818 & 2.2500 & 1.0777 & 2.2290\\ 3.3302 & 2.2075 & 1.0695 & 2.2232\\ 3.3359 & 2.1552 & 1.0696 & 2.3041\\ 3.3810 & 2.1221 & 1.0359 & 2.2912 \end{array}\right)$	$\left(\begin{array}{cccc} 3.4502 & 2.2955 & 1.0995 & 2.2741\\ 3.3975 & 2.2521 & 1.0911 & 2.2681\\ 3.4034 & 2.1987 & 1.0912 & 2.3507\\ 3.4494 & 2.1650 & 1.0568 & 2.3375 \end{array}\right)$

## Data Availability

The data presented in this study are openly available in https://www.kaggle.com/berkeleyearth/climate-change-earth-surface-temperature-data (accessed on 25 September 2024).
